# Air Pollution and Health: The Need for a Medical Reading of Environmental Monitoring Data

**DOI:** 10.3390/ijerph17072174

**Published:** 2020-03-25

**Authors:** Marcello Iriti, Prisco Piscitelli, Eduardo Missoni, Alessandro Miani

**Affiliations:** 1 Italian Society of Environmental Medicine (SIMA), 20133 Milan, Italy; marcello.iriti@unimi.it (M.I.); alessandro.miani@gmail.com (A.M.); 2 Department of Agricultural and Environmental Sciences, University of Milan, 20133 Milan, Italy; 3 Euro Mediterranean Scientific Biomedical Institute (ISBEM), Rue de Bellard 20, 1040 Bruxelles, Belgium; 4 Department of Sociology and Social Research, Bicocca University, 20133 Milan, Italy; eduardomissoni@gmail.com; 5 Centre for Research on Health and Social Care Management (CERGAS), SDA Bocconi School of Management, 20133 Milan, Italy; 6 Department of Environmental Science and Policy, University of Milan, 20133 Milan, Italy

**Keywords:** air pollution, health protection, particulate matter, air quality standards

## Abstract

Air pollution is a recent public health issue. In 2006, the World Health Organization (WHO) published updated air quality guidelines for a number of air pollutants (including PM10 and PM2.5), which recommended for particulate matter annual average concentration levels at half or less the limit values set by European legislation. In the European Union, around 80% of the European urban population is exposed to air pollution above the levels recommended by the WHO guidelines. Only in 2015 the WHO addressed for the first time the topic of the health impacts of air pollution in its general assembly, which adopted a resolution clearly defining air pollution as the world’s largest single environmental health risk factor. Nowadays, the WHO considers air pollution as a major public health threat, causing a 7% increase in overall mortality for each increase of 10 μg/m^3^ in annual average of PM2.5. This result has been achieved thanks to the outstanding efforts of the director of the WHO’s Environment and Public Health Department, Dr. Maria Neira, who has devoted her full commitment to highlighting the consequences that air pollution has on people’s health. More recently, at European level, the Air Quality Directive has been subject to a fitness check, published in 2019; the European Green Deal has since announced its aim to align EU air quality standards more closely with the WHO recommendations. Every year, the European Environment Agency (EEA) publishes its “Air Quality in Europe” Report to assess the figures on air pollution across Europe and related health impacts. However, environmental data provided by official regional or national agencies—used by decision makers to adopt preventive measures such as limitations on urban traffic or domestic heating—refer to legal thresholds established by the law (usually on the basis of values set at European level, at least for the EU). These legal thresholds, however, are not adequate to fully protect population against all impacts from air pollution as recommended by WHO and scientific evidence. Therefore, we point out the need for a medical reading of environmental monitoring data that should be performed both at national and regional or local level by health authorities, to foster population health protection against air pollution and guarantee the application of the precautionary principle. A stronger cooperation between environmental agencies and health authorities is needed to address the new challenges to human and planetary health arising from air pollution and climate change. Health authorities should integrate their medical staff with new professionals and researchers with adequate training in environmental sciences to foster population health protection against air pollution. For this purposes, multi-disciplinary research units or teams should be established by local health authorities on environmental health topics, working together with medical staff and environmental agencies for a mutual integration of competencies.

## 1. Air Pollution and Health: ARecent Issue

In 2006, the World Health Organization (WHO) published updated Air Quality Guidelines for a number of air pollutants, which recommend for particulate matter annual average concentration levels at half or less the limit values set by European legislation [1]. According to these air quality guidelines, annual average concentrations of PM10 should not exceed 20 μg/m^3^ (compared to the current limit value set by the EU of 40 μg/m^3^) and PM2.5 should not exceed 10μg/m^3^ (compared to the current EU limit value, set by EU legislation at 25 μg/m^3^) [1]. A number of countries have already adopted air quality standards for annual averages of PM10 and PM2.5 that are closer to the 2005 WHO guidelines (including Switzerland, Canada, Norway, Japan, and the United States of America). In the European Union, around 80% of the European urban population is exposed to air pollution above the levels recommended by the WHO guidelines [2].The World Health Organization has furthermore noted that the adverse effects on health of particulate matter are well documented, and that there is no evidence of a safe level of exposure or a threshold below which no adverse health effects occur, including exacerbations of respiratory diseases (e.g., asthma, especially in children). 

In 2013, the International Agency for Research on Cancer (IARC) had already classified outdoor air pollution in general, and particulate matter in particular, as *carcinogenic to humans* (IARC Class 1), pointing out that fine dusts are known to produce severe health impacts even at very low concentrations [3]. Surprisingly, only on 26 May 2015 the World Health Organization addressed for the first time the topic of the health impacts of air pollution, and its general assembly adopted a resolution clearly defining air pollution as the world’s largest single environmental health risk factor. Nowadays, WHO considers air pollution a major public health threat, causing a 7% increase in overall mortality for each increase of 10 μg/m^3^ in annual average of PM2.5 [4]. This result has been achieved thanks to the outstanding efforts of the director of the WHO’s Environment & Public Health Department, Dr. Maria Neira, who has devoted her full commitment to highlighting the consequences that air pollution has on people’s health. More recently, at European level, the Air Quality Directive has been subject to a fitness check, published in 2019; the European Green Deal has since announced its aim to align EU air quality standards more closely with WHO recommendations, but the cut to the limits on PM10 and PM 2.5 has not yet been accomplished.

## 2. Air Pollution: An under-Perceived Threat For public Health and Society

The European Environment Agency (EEA) publishes, every year, a specific Air Quality Report concerning 41 European countries (including the EU Member States). The most recent report was issued in autumn 2019, and again recognizes air pollution as a significant threat to human health in European cities, resulting in considerable impacts in terms of premature mortality, medical costs, and loss of productivity [2]. According to the EEA’s “Air Quality in Europe—2019 Report”, the most harmful pollutants for human health are particulate matter (PM), nitrogen dioxide (NO_2_) and ground-level ozone (O_3_), resulting in about 538,000 estimated premature deaths across Europe and almost 506,000 in the 28 countries belonging to the European Union, including the UK (Table 1). As a proxy for the economic impact of air pollution, we can refer to the indicator “years of life lost” (YLL) attributable to air pollution via PM2.5 and NO_2_ and O_3_ exposure, which has been estimated by the EEA at about 4,150,000 for Europe as whole and 4,466,000 for the 28 EU member States (Table 1). If we consider that, as Chiabai, Spadaro and Neuman have recently assessed [5], each year of life lost for European people corresponds to an annual value of 100,000 euros, it comes up more clearly how huge is the impact of air pollution both in terms of population health and economic consequences: more than four billion euros per year only for this indicator. A recent fitness check of the EU’s air quality legislation also confirmed the substantial economic impact of air pollution. 

This evidence demonstrates that individual health risks deriving from the inhalation of air pollutants are likely under-perceived, as well as the related societal and economic burden due to premature deaths and years of life lost. It took decades to define the risks related to cigarette smoking, based on scientific evidence, but the perception of individual risk arising from smoking is still not clear to smokers. One might argue that the same is happening with the issue of health consequences of air pollution. The fact that risk perception in the public opinion concerning this dramatic topic is largely inadequate is confirmed by figures provided by the EEA, which show that WHO guideline values for fine particulate matter (PM2.5) are exceeded at 69% of all reporting monitoring stations; as a result, 77% of the urban population across the EU was exposed to air pollution atlevels indicated as harmful by the World Health Organization [1,2]. Indeed, the recent fitness check of the air quality legislation of the EU highlights that current EU air quality standards are not as ambitious as established scientific advice suggests for several air pollutants, especially fine particulate matter (PM2.5). The European Green Deal has announced that the Commission will strive to steer Europe towards a zero-pollution ambition and, in that context, draw on the lessons learnt from the evaluation of the current air quality legislation, with a view to revise EU air quality standards and align them more closely with the WHO recommendations. 

## 3. The Need for a Medical Reading of Environmental Monitoring Data

A huge number of reliable epidemiological studies have associated air pollution with increased mortality due to all causes in the general population, especially cardio-cerebral-vascular accidents and respiratory diseases or lung cancers [6,7,8,9,10,11,12,13,14,15,16,17,18,19,20,21]. Moreover, an increase in the number of hospitalizations has been proved within 48–72 hours from peak concentrations of fine particulate matter [22,23,24,25,26,27,28,29]. Long term negative effects of air pollution have also been documented for neurodegenerative diseases as well as in terms of potentiality to cause type-2 diabetes and leukemia [30,31,32]. Scientific evidence indicates that all the health effects due to PM 2.5 and PM 10 are already displayed at the current limits of particulate concentrations allowed in EU Member States, both in terms of all-cause mortality and cause-specific mortality (i.e., cardiopulmonary diseases and lung cancer) [21]. However, environmental data provided by official regional or national agencies—used by decision makers to adopt preventive measures such as limitations on urban traffic or domestic heating—are still referred to air quality standards established by the law (usually on the basis of standards set at European level, at least for the EU).These legal thresholds, however, are not fully in line with the levels recommended by the WHO to protect the population against all impacts from air pollution, particularly children or more vulnerable people [24]. A stronger cooperation between environmental agencies and health authorities is needed to address the new challenges to human and planetary health arising from air pollution and climate change. Finally, it is important to provide, in a systematic way, a medical reading of environmental monitoring data, and guarantee the application of the precautionary principle enshrined in Article 191 of the Treaty on the Functioning of the European Union and in the communication of 22 February 2000 of the European Commission. Health authorities, both at national and regional or local level, should integrate their medical staff with new professionals and researchers adequately trained in environmental sciences to foster population health protection against air pollution. For this purposes, multi-disciplinary research units or teams should be established by local health authorities on environmental health topics, working together with medical staff and environmental agencies for a mutual integration of competencies. 

## Figures and Tables

**Table 1:** Premature deaths and years of life lost (YYL) attributable to PM_2.5_, NO_2_ and O_3_ exposure in 41 European countries and the EU-28. Data concerning year 2018 (modified from EEA 2019 Air Quality Report)^2^.

Country	Population (1000)	Premature Deaths Due to PM_2.5_	*YYL* *Due to PM2.5*	Premature Deaths Due to NO_2_	*YYL* *Due to NO_2_*	Premature Deaths Due to O_3_	*YYL Due to O_3_*
Austria	8700	5300	*60,200*	1000	*12,200*	270	*4000*
Belgium	11,311	7600	*77,600*	1600	*16,200*	180	*2400*
Bulgaria	7154	13,100	*142,000*	1100	*6400*	280	*3700*
Croatia	4191	5300	*46,900*	260	*4500*	190	*2500*
Cyprus	1184	580	*7400*	240	*300*	30	*410*
Czechia	10,554	9600	*105,500*	240	*5100*	350	*5000*
Denmark	5707	2700	*30,100*	80	*860*	90	*980*
Estonia	1316	500	*6300*	<1	*40*	20	*230*
Finland	5487	1500	*16,000*	<1	*470*	60	*570*
France	64,977	33,200	*414,700*	7500	*112,400*	1400	*21,600*
Germany	82,176	59,600	*638,500*	11,900	*134,200*	2400	*31,800*
Greece	10,784	12,900	*120,700*	2900	*23,100*	640	*6400*
Hungary	9830	12,100	*139,300*	770	*14,300*	380	*6000*
Ireland	4726	1100	*12,000*	50	*310*	30	*230*
Italy	60,666	58,600	*593,700*	14,600	*200,700*	3000	*32,100*
Latvia	1969	1700	*17,600*	60	*1400*	60	*600*
Lithuania	2889	2600	*27,400*	20	*760*	70	*940*
Luxembourg	576	230	*2700*	50	*510*	10	*110*
Malta	450	210	*2700*	<1	*180*	20	*180*
Netherlands	16,979	9200	*103,800*	1500	*19,900*	270	*3300*
Poland	37,967	43,100	*533,800*	1500	*20,400*	1100	*3300*
Portugal	9809	4900	*56,300*	610	*9100*	320	*16,600*
Romania	19,761	23,400	*271,600*	2600	*14,100*	490	*3200*
Slovakia	5426	4800	*59,900*	20	*2700*	160	*2600*
Slovenia	2064	1700	*20,000*	70	*1800*	70	*1100*
Spain	44,145	24,100	*290,500*	7700	*92,400*	1500	*19,100*
Sweden	9851	2900	*28,300*	30	*1000*	120	*1400*
UK	65,379	31,800	*324,900*	11,800	*99,700*	530	*6400*
Albania	2876	5100	*14,500*	70	*1300*	180	*6890*
Andorra	73	40	*540*	<1	*40*	<5	*40*
Bosnia and Herzegovina	3516	5400	*41,700*	20	*1700*	120	*2000*
Iceland	333	60	*670*	<1	*30*	<5	*5*
Kosovo	1772	3800	*36,300*	20	*650*	100	*1300*
Liechtenstein	38	20	*210*	<1	*30*	<5	*20*
Monaco	38	30	*290*	10	*270*	<5	*20*
Montenegro	622	630	*7300*	<1	*260*	20	*410*
North Macedonia	2071	3400	*30,400*	110	*1200*	70	*1100*
Norway	5211	1300	*12,900*	130	*2000*	50	*550*
San Marino	33	30	*280*	<1	*10*	<5	*20*
Serbia	7076	13,700	*127,800*	1500	*1796*	280	*4300*
Switzerland	8327	3700	*42,800*	620	*10,500*	240	*3300*
**EU-28**	**506,028**	**374,000**	***4,150,000***	**68,000**	***795,000***	**14,000**	***180,000***
**Total**	**538,014**	**412,000**	***4,446,000***	**71,000**	***821,000***	**15,100**	***193,800***
